# A case study on the implementation of quality and comprehensive indicators set in routine diabetes care

**DOI:** 10.1186/s13690-024-01348-8

**Published:** 2024-08-09

**Authors:** Ana Cristina García-Ulloa, José Luis Cárdenas-Fragoso, Diana Hernández-Juárez, Nancy Haydée Serrano-Pérez, Paula Blancarte-Jaber, Carlos Aguilar-Salinas, Nancy R. Mejía-Domínguez, Alejandro Zentella-Dehesa, Sergio Hernández-Jiménez, for the Group of Study CAIPaDi, María Teresa Alcántara-Garcés, Adriana Siboney Araujo González, Denise Liliana Arcila-Martínez, Rodrigo Arizmendi-Rodríguez, Humberto Del Valle-Ramírez, Arturo Flores García, Mariana Granados-Arcos, Héctor Manuel Infanzón-Talango, Arely Hernández Jasso, María Victoria Landa-Anell, Claudia Lechuga-Fonseca, Marco Antonio Melgarejo-Hernández, Angélica Palacios-Vargas, Liliana Pérez-Peralta, Maureen Alexis Acosta Mireles, David Rivera de la Parra, Alejandra Estefanía Montserrat Rodríguez-Ramírez, Francis Evelyn Rojas-Torres, Sandra Sainos-Muñoz, Nancy Haydee Serrano-Pérez, Héctor Rafael Velázquez-Jurado, Andrea Liliana Villegas-Narváez, Luz Elena Urbina-Arronte, Francisco J. Gómez-Pérez, David Kershenobich-Stalnikowitz

**Affiliations:** 1 Centro de Atención Integral del Paciente Con Diabetes, Instituto Nacional de Ciencias Médicas y Nutrición Salvador Zubirán, Mexico City, Mexico; 2 Project Manager for Outcomes Research, International Consortium of Health Outcomes Measurements, London, England, UK; 3 Dirección de Investigación, Instituto Nacional de Ciencias Médicas y Nutrición Salvador Zubirán, Mexico City, Mexico; 4 Red de Apoyo a La Investigación, Universidad Nacional Autónoma de México, Mexico City, Mexico; 5 Departamento de Medicina Genómica y Toxicología Ambiental, Instituto de Investigaciones Biomédicas Universidad Nacional Autónoma de México. Unidad de Bioquímica - Instituto Nacional de Ciencias Médicas y Nutrición Salvador Zubirán, Mexico City, Mexico

**Keywords:** Diabetes management, Patient-reported outcomes, Glycemic control, Blood pressure management, CAIPaDi model, ICHOM outcomes, Patient satisfaction

## Abstract

**Introduction:**

Type 2 diabetes is a major public health issue in Mexico due to its high prevalence and its projection for the coming years for this disease. Findings on multidisciplinary care related to chronic diseases have proven effective, based on measurement of patient-centered outcomes, The Center of Comprehensive Care for Patients with Diabetes (CAIPaDi) is a multidisciplinary program focused on reducing diabetes complications. This case study aims to illustrate the results of implementing health outcomes measurements and demonstrate the beneficial effects of establishing a comprehensive model of care through a patient-centered approach.

**Methods:**

A descriptive analysis of the comprehensive care indicators of patients with type 2 diabetes treated in the CAIPaDi program between 2013 and 2023 was conducted. The results were structured according to the standard set of outcomes for diabetes proposed by the International Consortium for Health Outcomes Measurements (ICHOM).

**Results:**

The baseline and prospective registration of consultations was completed for five years, complying with 25 of the 26 indicators of the ICHOM set**.** In diabetes control, 56.5% of patients had A1c ≤ 7%, 87.9% had BP ≤ 130/80 mmHg, 60.9% had LDL-cholesterol < 100 mg/dl, and obesity rates decreased from 42.19% to 30.6% during annual consultations. Fewer years of diagnosis before the first visit is key to overall improvement in program adherence (*P* = 0.02).

In acute events, a hyperglycemic crisis occurred in only two cases and severe hypoglycemia episodes in 8 patients. For chronic complications, no lower limb amputations occurred. Cardiovascular outcomes occurred in < 1%. Periodontal disease was analyzed, and periodontitis decreased from 82.9% to 78.7%. Mortality reports were low, with COVID-19 being the main cause of death. Patient-reported outcomes demonstrated reductions in anxiety, depression, and diabetes distress during follow-up.

**Conclusion:**

Registering quality-of-care indicators is feasible in a comprehensive care program. It allows improving the medical, mental health, and lifestyle outcomes of patients with type 2 diabetes and provides relevant data for planning health programs. A quick diagnosis before program adherence is crucial for overall improvement in patients.

**Table:** 

**Textbox 1. Contributions to the literature**	
• A multidisciplinary approach should be considered to provide effective, high-quality treatment and decrease the risk of long-term complications. • Clinical and biochemical values must be considered, and mental health and patient-reported outcomes need to be integrated into daily clinical decisions. • Using a standardized set of outcomes within a multidisciplinary approach may aid in achieving adequate metabolic treatment goals.	

## Background

Projections in diabetes estimate that by 2045, over 783 million people will be living with diabetes globally [1]. The disease burden is a matter of public health, given that in 2030, diabetes will account for the seventh cause of death worldwide [2]. Type 2 diabetes (T2D) is highly prevalent in Mexico. The National Health Surveys in Mexico have shown that the prevalence of type 2 diabetes mellitus increased from 7.5% in 2000 [3], 14.7% in 2018 [4], and 18.3% in 2021 [5]. This number is expected to rise to 21 million in 2045 [1]. From January to June 2023, T2D was the second leading cause of death in the Mexican adult population [6].

Optimized multidisciplinary diabetes care has proven effective in improving glycemic control and clinical outcomes [7, 8]. Reduction in diabetes incidence could potentially impact the costs of healthcare systems, maximizing resources [9]. Besides the traditional clinical and physiological outcomes, the outcomes of a clinical intervention obtained by a patient and patient-reported outcomes (PRO) provide valuable information. Patient-reported outcomes measures (PROM) may be generic (global assessments, such as health-related quality of life), domain-specific (measures regarding a specific domain, such as dyspnea), or a specific condition (for example, mental health comorbidities) [10].

In 2020, the International Consortium for Health Outcomes Measures (ICHOM) presented an initiative to establish a standard set of outcomes to ensure proper quality of diabetes care, specifically focusing on the patient's perspective. This effort has aided in implementing a consistently applied homogenous value-based care system. These outcomes can be categorized as clinical outcomes, health service utilization, survival outcomes, PRO, and PROM (Table 1) [11]. CAIPaDi (*Centro de Atención Integral del Paciente con Diabetes*) is a diabetes-oriented center focused on the wellbeing and quality of life of patients living with diabetes through outcomes measurement and a value-based healthcare approach [12, 13].

**Table 1:** Standard outcomes proposed by International Consortium for Health Outcomes Measurement (ICHOM)

Diabetes control	Glycemic control	HbA1c, with adequate control if < 7%
	Blood pressure	Adequate control if < 130/80 mmHg
	BMI	Classified as normal weight (18.5–24.9 m/kg^2^), overweight (25–29.9 m/kg^2^), or obesity (≥ 30 m/kg^2^)
	Lipid profile	LDL-c. Adequate control if < 100 mg/dL
Acute complications	Hyperglycemic crises	Development of diabetic ketoacidosis or hyperglycemic hyperosmolar state
	Hypoglycemia	Hypoglycemic events
	Severe hypoglycemia	A hypoglycemic event requiring assistance
	Acute cardiovascular events	Myocardial infarction, stroke
	Lower limb amputation	Presence of condition
Chronic complications	Autonomic neuropathy	Presence of condition (erectile dysfunction, neurogenic bladder, dizziness and fainting, urinary problems, sweating problems, sluggish pupil, exercise intolerance, constipation, and diarrhea)
	Peripheral neuropathy	Positive or negative neuropathic symptoms reported by patients They were assessed by monofilament and tuning fork
	Lower limb ulcerations	Presence of active ulcers
	Peripheral artery disease	They were evaluated with ankle-brachial-pressure index < 0.8
	Chronic kidney disease	Glomerular filtration rate < 60 mL/min/1.73m^2^ with CKD-EPI calculator and/or ACR ≥ 30 mg/g
	Cerebrovascular disease, ischaemic heart disease, or chronic heart failure	Presence of such a condition
	Periodontal health	Evaluation in the previous year
	Lipodystrophy	Presence of such a condition
Health services	Hospitalization	Number of visits in a year (in days)
	ER attendance	Number of visits in a year
Survival	Death	Cause of death
Patient-reported outcome measures	Psychological wellbeing	Captured using WHO-5
	Diabetes distress	It was captured using PAID
	Depression	Captured using PHQ-9

*Table adapted from ICHOM. Abbreviations: HbA1c* glycated hemoglobin, *BMI* body mass index, *LDL-c* low-density lipoprotein-cholesterol, *CKD-EPI* Chronic Kidney Disease Epidemiology Collaboration, *ACR* albumin-creatinine ratio, *ER* emergency room, *PAID* Problem Areas in Diabetes questionnaire, *PHQ-9* Patient Health Questionnaire, *WHO-5* World Health Organisation questionnaire

This paper presents the application of the ICHOM set of person-centered outcomes in a comprehensive and multidisciplinary program for patients with diabetes.

## Methods

This is a case study.

Theoretical framework of the case study.

The CAIPaDi model is divided into interventions focused on medical care, diabetes education, lifestyle interventions, and mental health evaluations. These interventions aim to comprehensively evaluate and treat diabetes and comorbidities, train the patient for self-care, and prevent disabling complications.

It comprises two phases. The first phase consists of four visits, each one month apart. The second phase encompasses annual evaluations. In every visit, blood and cabinet tests and urine samples are performed. Nine specialists evaluate the patients every time, including endocrinologists, optometrists/ophthalmologists, diabetes educators, nutritionists, dentists, psychologists and psychiatrists, foot care specialists, and exercise specialists [12, 13]. All specialists registered indicators specific to each area, according to the ICHOM set.

Since the nine specialists in each visit attend all patients, the CAIPaDi program gave 91,062 consultations over the five years, of which 10,118 were about Diabetes education.

### Selection criteria

We conducted a descriptive analysis of patients enrolled between October 31, 2013, and June 30, 2023. The sampling method was a non-probability, convenience sampling. Inclusion criteria consisted of adult patients with T2D with less than five years of diagnosis, non-smokers, and without disabling chronic complications such as chronic kidney disease, ischaemic heart disease, cerebrovascular disease, limb amputations, or severe visual impairment. The CAIPaDi program was originally designed to attend patients with type 2 diabetes due to the high prevalence that exists in our country. Likewise, it was initially considered to attend patients with less than 5 years of diagnosis since the program has a preventive approach to the chronic complications of diabetes. Exclusion criteria were a mental or psychiatric illness that prevents them from learning and following instructions, chronic complications of the disease, patients undergoing treatment for any cancer, pregnant women, alcohol consumption that interferes with their work or social activities, an illness that requires surgery or prevents carrying out physical activity, and patients with BMI > 45 kg/m^2^.

### Outcomes measurement

Relevant indicators of each intervention have been recorded using SMID (Comprehensive Diabetes Monitoring System) software, which is designed to have a checklist to comply with in each consultation. According to the standard outcomes proposed by ICHOM, several items were evaluated in our study regarding diabetes control, acute events, chronic complications, health services, survival, and patient-reported outcome measures, as seen in Table 2.

**Table 2:** Summary by sex and age group for International Consortium for Health Outcomes Measurement (ICHOM) diabetes control variables. Percentages shown in parenthesis (%)

	**Level**	**Sex**	**Age group**	**Basal** **n = 3241**	**3 months** **n = 2295**	**1 year** **n = 1492**	**2 years** **n = 1005**	**3 years** **n = 663**	**4 years** **n = 388**	**5 years** **n = 204**
**HbA1c**	≤ *7%*	Men	< 40 years	70 (2.16)	103 (4.49)	47 (3.15)	22 (2.19)	15 (2.26)	5 (1.29)	2 (0.98)
			40–60 years	297 (9.16)	505 (22.00)	268 (17.96)	163 (16.22)	106 (15.99)	62 (15.98)	31 (15.20)
			> 60 years	122 (3.76)	167 (7.28)	91 (6.10)	63 (6.27)	51 (7.69)	33 (8.51)	18 (8.82)
		Women	< 40 years	76 (2.34)	84 (3.66)	35 (2.35)	20 (1.99)	17 (2.56)	10 (2.58)	4 (1.96)
			40–60 years	510 (15.74)	704 (30.68)	354 (23.73)	222 (22.09)	140 (21.12)	84 (21.65)	39 (19.12)
			> 60 years	195 (6.02)	231 (10.07)	158 (10.59)	105 (10.45)	73 (11.01)	32 (8.25)	18 (8.82)
	> *7%*	Men	< 40 years	164 (5.06)	29 (1.26)	25 (1.68)	22 (2.19)	12 (1.81)	8 (2.06)	6 (2.94)
			40–60 years	620 (19.13)	140 (6.10)	157 (10.52)	124 (12.34)	84 (12.67)	43 (11.08)	19 (9.31)
			> 60 years	153 (4.72)	45 (1.96)	48 (3.22)	43 (4.28)	19 (2.87)	16 (4.12)	12 (5.88)
		Women	< 40 years	122 (3.76)	32 (1.39)	34 (2.28)	20 (1.99)	7 (1.06)	5 (1.29)	0 (0)
			40–60 years	737 (22.74)	200 (8.71)	214 (14.34)	156 (15.52)	113 (17.04)	73 (18.81)	47 (23.04)
			> 60 years	175 (5.40)	55 (2.40)	61 (4.08)	45 (4.48)	26 (3.92)	17 (4.38)	8 (3.92)
**BP**	≤ *130/80 mmHg*	Men	< 40 years	132 (4.07)	99 (3.82)	55 (3.69)	33 (3.28)	20 (3.02)	10 (2.58)	NA
			40–60 years	483 (14.90)	470 (18.15)	277 (18.57)	198 (19.70)	127 (19.16)	75 (19.33)	NA
			> 60 years	140 (4.32)	140 (5.41)	88 (5.90)	68 (6.77)	40 (6.03)	31 (7.99)	NA
		Women	< 40 years	133 (4.10)	98 (3.79)	58 (3.89)	33 (3.28)	20 (3.02)	14 (3.61)	NA
			40–60 years	731 (22.55)	754 (29.12)	423 (28.35)	286 (28.46)	182 (27.45)	115 (29.64)	NA
			> 60 years	195 (6.02)	205 (7.92)	165 (11.06)	105 (10.45)	76 (11.46)	36 (9.28)	NA
	> *130/80 mmHg*	Men	< 40 years	102 (3.15)	33 (1.27)	17 (1.14)	11 (1.09)	7 (1.06)	3 (0.77)	6 (2.94)
			40–60 years	434 (13.39)	470 (18.15)	148 (9.92)	89 (8.86)	63 (9.50)	30 (7.73)	32 (15.69)
			> 60 years	135 (4.17)	72 (2.78)	51 (3.42)	38 (3.78)	30 (4.52)	18 (4.64)	21 (10.29)
		Women	< 40 years	65 (2.01)	18 (0.70)	11 (0.74)	7 (0.70)	4 (0.60)	1 (0.26)	4 (1.96)
			40–60 years	516 (15.92)	150 (5.79)	145 (9.72)	92 (9.15)	71 (10.71)	42 (10.82)	55 (26.96)
			> 60 years	175 (5.40)	80 (3.09)	54 (3.62)	45 (4.48)	23 (3.47)	13 (3.35)	17 (8.33)
**BMI**	*Normal weight*	Men	< 40 years	40 (1.23)	13 (0.57)	9 (0.60)	4 (0.40)	2 (0.30)	0	0
			40–60 years	147 (4.54)	104 (4.53)	52 (3.49)	40 (3.98)	26 (3.92)	13 (3.35)	9 (4.41)
			> 60 years	66 (2.04)	59 (2.57)	37 (2.48)	30 (2.99)	19 (2.87)	16 (4.12)	12 (5.88)
		Women	< 40 years	42 (1.30)	27 (1.17)	16 (1.07)	7 (0.70)	2 (0.30)	3 (0.77)	2 (0.98)
			40–60 years	189 (5.83)	169 (7.35)	86 (5.76)	55 (5.47)	48 (7.24)	35 (9.02)	13 (6.37)
			> 60 years	70 (2.16)	70 (3.05)	45 (3.02)	34 (3.38)	26 (3.92)	13 (3.35)	7 (3.43)
	*Overweight*	Men	< 40 years	77 (2.38)	56 (2.44)	31 (2.08)	21 (2.09)	17 (2.56)	9 (2.32)	5 (2.45)
			40–60 years	405 (12.50)	293 (12.75)	207 (13.87)	146 (14.53)	95 (14.33)	57 (14.69)	22 (10.78)
			> 60 years	131 (4.04)	103 (4.48)	65 (4.36)	50 (4.98)	36 (5.43)	24 (6.19)	15 (7.35)
		Women	< 40 years	71 (2.19)	45 (1.96)	27 (1.81)	16 (1.59)	12 (1.81)	5 (1.29)	2 (0.98)
			40–60 years	479 (14.78)	385 (16.75)	244 (16.35)	175 (17.41)	98 (14.78)	66 (17.01)	40 (19.61)
			> 60 years	159 (4.91)	127 (5.53)	91 (6.10)	63 (6.27)	40 (6.03)	21 (5.41)	11 (5.39)
	*Obesity*	Men	< 40 years	117 (3.61)	63 (2.74)	32 (2.14)	19 (1.89)	8 (1.21)	4 (1.03)	3 (1.47)
			40–60 years	365 (11.26)	249 (10.84)	166 (11.13)	101 (10.05)	69 (10.41)	35 (9.02)	19 (9.31)
			> 60 years	78 (2.41)	50 (2.18)	37 (2.48)	26 (2.59)	15 (2.26)	9 (2.32)	3 (1.47)
		Women	< 40 years	85 (2.62)	45 (1.96)	26 (1.74)	17 (1.69)	10 (1.51)	7 (1.80)	0
			40–60 years	579 (17.86)	351 (15.27)	238 (15.95)	148 (14.73)	107 (16.14)	56 (14.43)	33 (16.18)
			> 60 years	141 (4.35)	89 (3.87)	83 (5.56)	53 (5.27)	33 (4.98)	15 (3.87)	8 (3.92)
**LDL-Cholesterol**	≤ *100 mg/dL*	Men	< 40 years	106 (3.27)	102 (4.44)	36 (2.41)	25 (2.49)	14 (2.12)	11 (2.84)	5 (2.45)
			40–60 years	363 (11.21)	480 (20.92)	221 (14.81)	145 (14.46)	109 (16.52)	65 (16.75)	32 (15.69)
			> 60 years	101 (3.12)	165 (7.19)	73 (4.89)	62 (6.18)	33 (5.00)	29 (7.47)	16 (7.84)
		Women	< 40 years	80 (2.47)	71 (3.09)	29 (1.94)	13 (1.30)	9 (1.36)	6 (1.55)	2 (0.98)
			40–60 years	403 (12.45)	635 (27.67)	250 (16.76)	182 (18.15)	127 (19.24)	100 (25.77)	48 (23.53)
			> 60 years	119 (3.68)	202 (8.80)	103 (6.90)	79 (7.88)	53 (8.03)	28 (7.22)	14 (6.86)
	> *100 mg/dL*	Men	< 40 years	128 (3.95)	30 (1.31)	36 (2.41)	19 (1.89)	13 (1.97)	2 (0.52)	3 (1.47)
			40–60 years	552 (17.05)	164 (7.15)	204 (13.67)	141 (14.06)	80 (12.12)	40 (10.31)	18 (8.82)
			> 60 years	174 (5.37)	47 (2.05)	66 (4.42)	44 (4.39)	35 (5.30)	20 (5.15)	14 (6.86)
		Women	< 40 years	118 (3.64)	45 (1.96)	40 (2.68)	27 (2.69)	15 (2.27)	9 (2.32)	2 (0.98)
			40–60 years	843 (26.03)	270 (11.76)	318 (21.31)	195 (19.44)	126 (19.09)	57 (14.69)	38 (18.63)
			> 60 years	251 (7.75)	84 (3.66)	116 (7.77)	71 (7.08)	46 (6.97)	21 (5.41)	12 (5.88)

*Abbreviations: HbA1c* glycated hemoglobin, *BP* blood pressure, *LDL-c* low-density lipoprotein- cholesterol, *BMI* body mass index, *DKA* diabetic ketoacidosis, *HHS* hyperglycemic, hyperosmolar state, *UTI* urinary tract infection, *CV* cardiovascular, *PAD* peripheral artery disease, *IHD* ischaemic heart disease, *CHF* chronic heart failure, *CKD* chronic kidney disease

#### Overview of the ICHOM parameters and their relevance to evaluating healthcare interventions

All sets of patient-centered outcome measures created by ICHOM are developed using the following parameters: clinical outcomes, patient-reported outcomes, outcome measures, case-mix variables, and time points. In the Diabetes Set, clinical outcomes cover diabetes control, survival, chronic complications, and acute events. Patient-reported outcomes address quality of life, symptom burden, and functional status; these are “directly reported by the patient without interpretation of the patient’s response by the clinician or anyone else” [14]. Outcome measures are the tools selected to measure each outcome in the categories defined within each parameter. Patient-reported outcome measures (PROMs) will measure patient-reported outcomes. Case-mix variables represent any factor related to the patient and/or the clinical condition, including its treatment, which may impact any outcome. Finally, through a standardized timeline, outcomes and case-mix variables can be measured based on the different time points indicated for each group of outcomes or variables specifically.

The importance of including and meeting all the aforementioned parameters lies, first and foremost, in measuring outcomes through a patient-centered perspective, therefore focusing on outcomes that matter to patients and improving their overall experience previous to, during, and post-treatment. Further, they allow healthcare providers to standardize outcomes measurement for all patients living with a given disease. When healthcare providers base their decision-making, define processes, and evaluate quality and performance based on uniform measures, comparability across providers across institutions, regions, and even countries becomes possible and more meaningful. As a result, there is more space for benchmarking and healthcare improvement, as providers can evaluate their performance compared to others and identify areas of opportunity for improvement.

#### Application of ICHOM Parameters

- Evaluation of CAIPaDi program outcomes based on each selected ICHOM parameter**.**

The ICHOM Set defines health-related quality of life, including psychological wellbeing, depression, and diabetes distress as the PROs most relevant to patients. The CAIPaDi program includes the use of the Diabetes Quality of Life (DQoL), the Hospital Anxiety and Depression (HADS), and the Problem Areas in Diabetes Questionnaire (PAID-20) PROMs to measure said outcomes, respectively. Clinical outcomes in the set include survival, disease control, chronic complications, health service utilization, and acute events. The CAIPaDi program measured the aforementioned outcomes as follows: survival is measured through vital status; disease control is measured through glycemic control, both through fasting glucose and HbA1c levels, blood pressure control, and lipid profile control; chronic complications are measured through treatment complications, nervous system complications, and micro and macrovascular complications which translate in cases of amputation, nephropathy, dialysis, and cardiovascular events, respectively; healthcare services utilization is measured through days of hospitalization related and number of emergency room visits, related to the disease; and finally, acute events are measured through episodes of diabetic ketoacidosis, hyperglycemic hyperosmolar syndrome, and severe hypoglycemic episodes.

The ICHOM Set requires at least two follow-up visits in each patient's first year of treatment, during which PROs will be measured in each visit, CROs will be measured at least annually, and case-mix variables will be measured. The CAIPaDi program includes at least four follow-up visits during the first year and at least one annual follow-up visit subsequently. This meets and exceeds the timeline indicated in the ICHOM Set.

- Presentation of quantitative data, such as changes in patient-reported outcomes, healthcare utilization metrics, and cost-effectiveness measures.

Based on the data collected through PROMs included in the CAIPaDi program previously mentioned, after 9 follow-up visits, the patients whose results indicated depression nearly halved, as well as those whose results indicated anxiety. Similarly, the number of patients who presented scores indicating "emotional burnout" lowered by almost 75% by the first year of treatment through this program. The overall quality of life decreased initially during the first months of treatment but later stabilized and remained at an average of 72 points through the DQoL tool between three months and five years of follow-up. Further, an estimated US $7264 per patient was saved throughout nine visits in five years, on average, complication-related emergency hospital visits, hospitalization, and treatment [15].

### Statistical analysis

This is a descriptive study. Data are presented as mean (± SD) or median and interquartile ranges (25–75) if they followed or did not have a normal distribution, respectively; according to the Kolmogorov–Smirnov test, percentages were used for discrete values.

For the analysis, we included 3241 patients at the initial visit, 2295 patients after three months of entering the program, 1492 patients at the first annual consultation, 1005 patients at the second annual visit, 663 patients at the third annual visit, 388 patients at the fourth annual visit and 204 patients after a 5-year follow-up. The decrease in sample size is due to abandonment, or some patients still need to arrive at their next evaluation visit. Likewise, given that some patients started treatment in later years, they have yet to reach five years of follow-up.

To examine the effect of adherence to the program on health improvement, we evaluated the positive chance of diabetes control variables using McNemar's test during the two follow-up periods. First, from the initial visit to the 5-year follow-up, and second, from the visit after three months to the 5-year follow-up. For the McNemar test, we constructed a "paired" contingency table for two visits involving the improvement/non-improvement of glycated hemoglobin, blood pressure, low-density lipoprotein-cholesterol, and BMI. Positive change or improvement was defined as hemoglobin ≤ 7%, blood pressure ≤ 130/80 mmHg, LDL-c ≤ 100 mg/dl, and no obesity. For BMI categories, we evaluated the improvement in 5-year follow-up in two forms: 1) obese vs. non-obese patients and 2) normal weight vs. non-normal weight patients. Finally, we defined overall improvement (presence/absence) as a successful glycated hemoglobin ≤ 7% and improvement in any of the other three performance variables (blood pressure ≤ 130/80 mmHg, LDL-c ≤ 100 mg/dl, and non-obesity). We performed a logistic regression analysis to examine the effects of demographic and psychological variables on overall improvement.

## Results

At the beginning of visit one (3241 patients) 56.3% were women, the average age was 55.8 ± 10.5 (18–78) and 40.4% were patients with less than one year of diabetes diagnosis. Regarding education, 0.3% were illiterate, 0.4% knew how to read and write, and 98.7 had some level of education. Of them, 41.5% had a master's degree or doctorate.

Table 3 shows the ICHOM standardized clinical outcomes implemented in the CAIPaDi model over five years. Adherence was evaluated by improvement in metabolism, anthropometric, and mental health parameters. The overall improvement was affected only by the number of years of diagnosis before the first visit, and fewer years of diagnosis before the first visit had a greater probability of improvement (*p* = 0.02; Table 4).

**Table 3:** International Consortium for Health Outcomes Measurement (ICHOM) standardized clinical outcomes

			**Basal** **n = 3551** **(%)**	**3 months** **n = 2489** **(%)**	**1 year** **n = 1573** **(%)**	**2 years** **n = 1059** **(%)**	**3 years** **n = 754** **(%)**	**4 years** **n = 444** **(%)**	**5 years** **n = 248** **(%)**
**1. Diabetes control**	**HbA1c**	≤ *7%*	1461 (41.1)	1999 (80.3)	1048 (66.6)	649 (61.3)	470 (62.3)	267 (60.1)	140 (56.5)
		> *7%*	2090 (58.8)	490 (19.7)	525 (33.4)	409 (38.6)	284 (37.7)	177 (39.9)	108 (43.5)
	**BP**	≤ *130/80 mmHg*	2304 (64.8)	2114 (84.9)	1286 (81.8)	881 (83.2)	629 (83.4)	381 (85.8)	218 (87.9)
		> *130/80 mmHg*	1246 (35.1)	375 (15.1)	288 (18.3)	177 (16.7)	125 (16.6)	63 (14.2)	30 (12.1)
	**BMI**	*Normal weight*	587 (16.5)	481 (19.3)	253 (16)	379 (35.7)	139 (18.4)	92 (20.7)	55 (22.1)
		*Overweight*	1446 (40.7)	1086 (43.6)	704 (44.7)	499 (47.1)	337 (44.6)	206 (46.3)	117 (47.1)
		*Obesity*	1489 (41.9)	913 (36.6)	612 (38.9)	176 (16.6)	278 (36.8)	146 (32.8)	76 (30.6)
	**LDL-Cholesterol**	≤ *100 mg/dl*	1320 (37.2)	1822 (73.2)	762 (48.4)	535 (50.5)	410 (54.4)	276 (62.2)	151 (60.9)
		> *100 mg/dL*	2228 (62.7)	667 (26.8)	810 (51.5)	521 (49.2)	341 (45.2)	169 (38.0)	97 (39.1)
**2. Acute complications**	**DKA / HHS events**		0	0	0	2 (0.2)	0	0	0
	**Hypoglycemic events**		744 (29.4)	425 (18.5)	319 (21.4)	216 (21.5)	155 (23.3)	78 (20.1)	46 (22.5)
	**Severe hypoglycemia events**		0	3 (0.12)	3 (0.19)	2 (0.18)	0	0	0
	**Acute cardiovascular events**		0	1 (0.04)	0	0	0	1 (0.2)	1 (0.4)
	**Lower limb amputation**		0	0	0	0	0	0	0
**2. Chronic complications**	**Autonomic neuropathy**								
	-Cardiovascular		5 (0.14)	ND	0	2 (0.19)	0	0	0
	-Gastrointestinal		9 (2.5)	ND	2 (0.13)	1 (0.06)	1 (0.13)	3 (0.67)	3 (1.2)
	-Genitourinary		266 (7.5)	ND	43 (2.7)	26 (2.5)	9 (1.2)	3 (0.67)	3 (1.2)
	**Peripheral neuropathy**								
	Symptoms		169 (4.7)	ND	38 (2.4)	30 (2.8)	14 (1.8)	10 (2.2)	4 (1.6)
	Assesed by monofilament		85 (2.3)	ND	19 (1.2)	16 (1.51)	11 (1.4)	3 (0.67)	1 (0.4)
	Aseesed by tuning fork		761 (21.4)	ND	312 (19.8)	194 (18.3)	132 (17.5)	66 (14.8)	26 (10.4)
	**Charcot’s foot**		0	1	0	0	0	0	0
	**Lower limb ulcerations**		0	0	0	0	0	0	0
	**Peripheral artery disease** (ankle-brachial pressure index < 0.8)		19 (0.5)	ND	7 (0.4)	6 (0.5)	4 (0.5)	3 (0.6)	1 (0.4)
	**Cardiovascular event**		0	1 (0.04)	3 (0.1)	3 (0.2)	1 (0.1)	1 (0.2)	0
	**Chronic kidney disease**								
	-GFR < 60 mL/min/1.73 m^2^)		0	ND	22 (1.4)	20 (1.9)	21 (2.8)	14 (3.2)	9 (3.6)
	-ACR ≥ 30 mg/g		629 (17.7)	187 (7.5)	197 (12.5)	150 (14.2)	112 (14.6)	78 (17.6)	39 (15.7)
	**Vision**								
	-Visual impairment (acuity)		438 (12.3)	ND	241 (6.7)	195 (5.5)	159 (4.4)	155 (28.6)	60 (28.6)
	-Retinopathy		546 (17.7)	ND	263 (16.7)	199 (18.8)	126 (16.7)	56 (12.6)	32 (12.9)
	**Periodontal health**								
	-Gingivitis		308 (8.9)	130 (6.0)	109 (7.8)	68 (7.3)	54 (8.0)	24 (6.3)	7 (3.5)
	-Periodontitis		2863 (82.9)	1678 (72.7)	1015(72.7)	682 (63.5)	495 (73.9)	291 (76.6)	155 (78.7)
**3. Health services**	**Hospitalization**		0	79 (3.17)	61 (3.8)	40 (3.7)	30 (3.9)	12 (2.7)	12 (4.8)
**4. Survival**	**Death**		0	2 (0.08)	8 (0.5)	5 (0.4)	2 (0.2)	0	3 (1.2)
**5. Patient-reported outcome measures**	**Diabetes distress** **(**PAID-20, points)		38.2	15.0	17.8	16.1	15.0	14.1	13.4
	**Depression**		789 (22.2)	196 (7.9)	191 (12.1)	137 (12.9)	88 (11.7)	57 (12.8)	26 (10.5)
	**HADd ≥ 8**		1028 (28.9)	327 (13.1)	242 (15.4)	129 (12.2)	77 (10.2)	59 (13.3)	37 (14.9)
	**Anxiety**		786 (22.1)	244 (9.8)	195 (12.4)	121 (11.4)	103 (13.7)	60 (13.5)	37 (14.9)
	**HADa ≥ 8**		1585 (44.6)	459 (18.4)	372 (23.6)	253 (23.9)	160 (21.2)	96 (21.6)	50 (20.2)
	**Quality of Life (DqoL) (points)**		92.2	71.1	73.7	72.8	70.7	69.8	70.1

*Abbreviations: HbA1c* glycated hemoglobin, *BP* blood pressure, *LDL-c* low-density lipoprotein- cholesterol, *BMI* body mass index, *DKA* diabetic ketoacidosis, *HHS* hyperglycemic, hyperosmolar state, *UTI* urinary tract infection, *CV* cardiovascular, *PAD* peripheral artery disease, *IHD* ischaemic heart disease, *CHF* chronic heart failure, *CKD* chronic kidney disease

**Table 4:** Results of the univariate logistic regression models on overall improvement

Variable (levels)	OR	95% IC	*P*-value
Years of diagnosis before the first visit	0.80	0.66 – 0.97	0.02*
Quality of Life	0.98	0.97 – 1.00	0.060
PAID	0.99	0.98 – 1.00	0.63
Stressors	1.08	0.61 – 1.90	0.77
Anxious disorder	0.92	0.44 – 1.92	0.84
Anxiety symptoms			0.56
Category 2	1.50	0.63 – 3.77	
Category 3	0.80	0.28 – 2.20	
Depression symptoms			0.56
Category 2	1.50	0.63 – 3.77	
Category 3	0.83	0.28 – 2.20	
Sex	0.67	0.38 – 1.18	0.16
Age of ingress to program (years)	1.01	0.98 – 1.05	0.24
Age category of ingress to program			0.34
30–40	0.97	0.29 – 3.24	
> 40	1.54	0.43 – 5.54	
Schooling			0.46
Doctorate	3.4	0.44 – 7.24	
Master's Degree	0.94	0.36 – 2.42	
Professional	0.77	0.22 – 2.58	
Baccalaureate	0.93	0.32 – 2.70	
Technician	1.05	0.39 – 2.80	
Highschool	0.78	0.23 –2.51	
Primary school	1.11	0.32 – 3.92	
Can read	5.53 $\times$ 10^–8^	NA – 1–71 $\times$ 10^8^	
Analphabet	1.35 $\times$ 10^7^	4.44 $\times$ 10^–10^ – NA	
Non-specified	5.5 $\times$ 10^–8^	NA – 1.04 $\times$ 10^10^	

### Diabetes control

In the baseline evaluation, glycemic control was present in 39.2% of the patients. At three months, 78.2% of the patients achieved adequate diabetes control. The proportion of patients with HbA1c ≤ 7% changed to 54.9% after five years of follow-up. In addition, for patients whose follow-up for five years was a significant improvement in HbA1c levels from the visit to after three months to 5-year follow-up (*P* = 5.5 × 10^–15^), but from the initial visit to the 5-year follow-up, there was no significant improvement (*P* = 0.63). The percentage of patients with blood pressure (BP) measured in goals diminished from baseline (44%) over time, ranging from 23 to 27% from three months to four years. In the fifth year of follow-up, 204 patients remained, and 66.1% reported a BP of ≤ 130/80 mmHg. Likewise, for patients with a follow-up of five years, a significant improvement in blood pressure (BP ≤ 130/80 mmHg) was observed both from the initial visit to the 5-year follow-up and from the visit after three months to 5-year follow-up were significant improvement (*P* = 7.1 × 10^–14^, *P* = 9.4 × 10^–7^, respectively). At the 5-year follow-up, the percentage of patients with LDL cholesterol < 100 mg/dl was 60.9. In addition, for patients who follow up for five years, a significant improvement in LDL-cholesterol (LDL-cholesterol < 100 mg/dl) was observed both from the initial visit to the 5-year follow-up, there was significant improvement (*P* = 1.6 × 10^–5^) and from the visit to after three months to 5-year follow-up (*P* = 2.0 × 10^–5^). The proportion of patients with overweight remained unchanged upon follow-up (between 40%-46.9%). The proportion of obesity decreased from 42.1% at the initial visit to 32%-36% at the annual consultations. However, there was no significant improvement in any BMI category or period for patients who follow-up for five years.

**Fig. 1:**
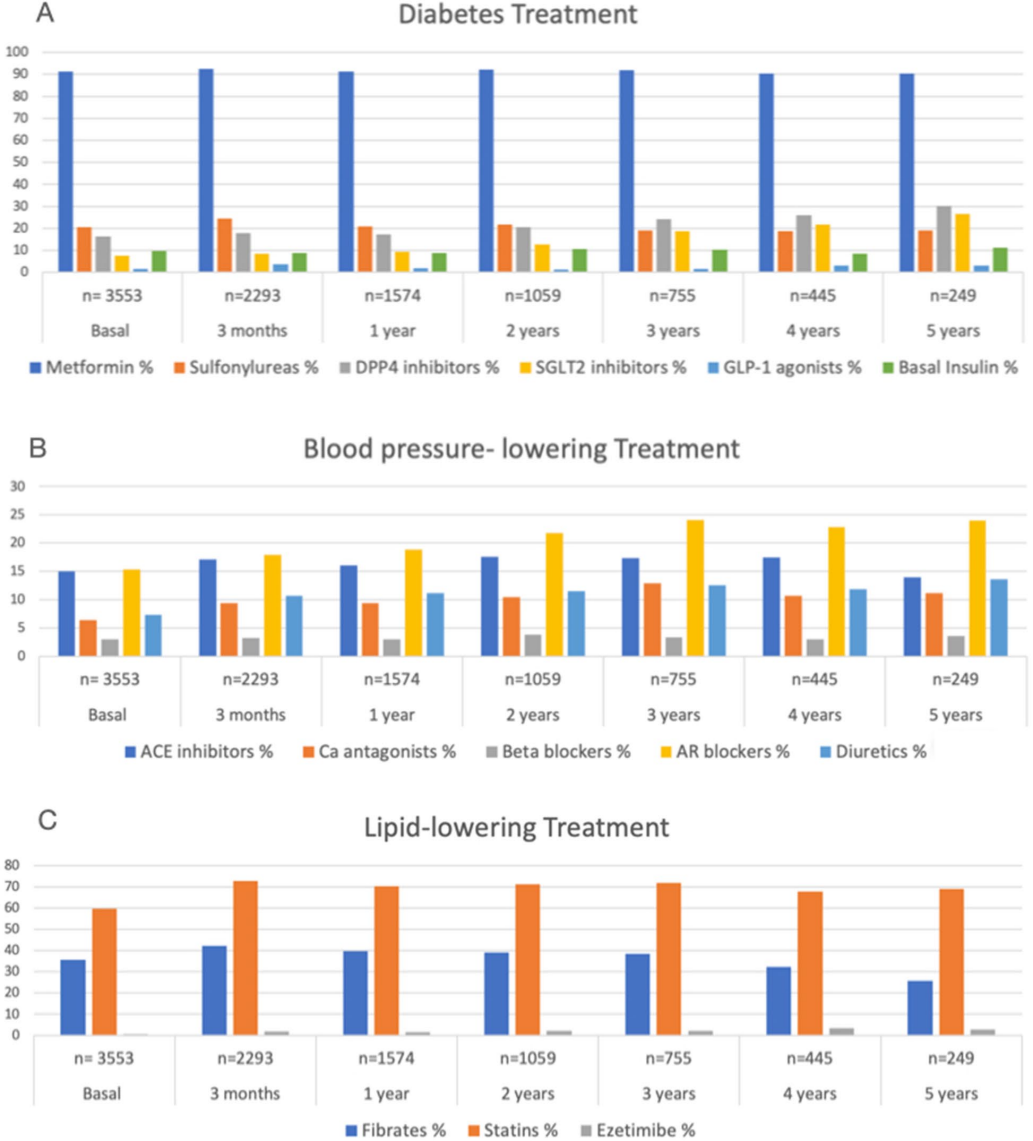
Treatment factor, Distribution and changes in percentages of treatments in the different visits of the CAIPaDi program. A: diabetes treatment; B: blood pressure treatment; C: lipid-lowering treatment. *Abbreviations:* ACE—angiotensin-converting enzyme, ARB - angiotensin receptor blocker, CA - calcium antagonists, DPP4i - dipeptidyl peptidase-4 inhibitors, GLP-1a - glucagon-like peptide-1 agonists, SGLT2i - sodium-glucose cotransporter-2 inhibitors

Some of the case mix variables included in the ICHOM set are related to treatment factors (prescribed medications and insulin use). Figure 1 shows the changes in treatment during the five years of follow-up. Through the years, we can see that some diabetes medications, like metformin, are often prescribed with a percentage of 90% or above in all the visits; the SGLT2 inhibitors and DPP IV inhibitors were increased in the prescriptions from a percentage of 5% and 14% respectively to 25% of both. Regarding the other groups, sulfonylureas are prescribed in many cases, mostly because of their low cost. Otherwise, the GLP-1 agonists remain with a low percentage (1–2.3%) because of their cost. Figure 1 shows the treatment distribution in each visit, with metformin being the most frequently prescribed treatment.

Relating to blood pressure-lowering therapy, ACE inhibitors and angiotensin receptor blockers are the most prescribed, followed by calcium antagonists and diuretics; the least prescribed are beta blockers because of their interactions with glucose control. Respecting lipid-lowering drugs, the statins kept their percentage through the visits (70%), and the prescription of fibrates decreased during follow-up.

### Acute events

Regarding acute complications, only one patient required hospitalization due to a hyperglycemic crisis. The highest proportion of hypoglycemia was noted at one year (23.3%), and this percentage kept decreasing afterward. Severe hypoglycemia, defined as hypoglycemia that required assistance, was recorded in less than 1%.

### Chronic complications

Chronic complications are important outcomes to measure and decrease in incidence. During the entire follow-up, we did not record any lower limb amputation. Peripheral artery disease, detected by abnormal ankle-brachial index (< 0.8), remained unchanged between 0.4%-0.6% during the follow-up. Regarding periodontal health, nearly two-thirds of our patients had periodontitis and, to a lesser degree, gingivitis. These percentages decreased from 8.9% to 3.% % for gingivitis and 82.9% to 78.7% for periodontitis. Since 2017, we have been registering the presence of lipodystrophy. During this time, we attended 3010 patients, and 51 had lipodystrophy (1.69%), 51 patients in the basal evaluation.

### Patient-reported outcome measures

Patient-reported outcomes evaluated three key aspects: psychosocial wellbeing, depression, and diabetes distress, that is, emotional distress attributable to a diabetes diagnosis.

Regarding anxiety and depression, nearly 30% of patients presented an overall score of 8 points or more upon their first visit for depression, and nearly 45% presented the same result for anxiety. When measuring the same outcomes upon visit 9, the percentage of patients with an overall score of 8 points or higher decreased to 14.92% for depression and 20.16% for anxiety. When measuring diabetes distress, i.e., emotional distress attributable to diagnosis and treatment for diabetes, through the PAID-20 questionnaire, 45.87% of patients presented a score of 40 or higher, indicating potential "emotional burnout" on their first visit. On the first annual visit, the percentage of patients with this score decreased to 11.76% and averaged 8% for the rest of the annual evaluations. Results for quality-of-life measurements through the DQoL tool showed an average of 92 points on the first visit, which decreased to 72 between three months and five years of follow-up. Results for the empowerment questionnaire showed an increase of approximately 9 points in the average score between basal evaluation and two months, from 73.8 to 82.2. This last score remained stable within the range of 81–84 points in the average score for the remainder of the visits. Self-efficacy results showed less variance, with an initial 87.2 score at three months, which was the first recording for this outcome, a decline to 83.6 at two years, and a gradual increase until reaching 86.1 at five years. These results are shown in Table 3.

Finally, patient satisfaction with care and overall experience was measured through a survey developed internally in CAIPaDi. When asked whether they were able to complete the questionnaires, i.e., PROMs, easily enough, 96% of patients who attended the consultation in person responded "Strongly agree" or "Agree"; no patients responded "Strongly disagree" and only 1% of patients responded, "Disagree." Regarding the same question, 97% of patients who attended consultation through video call or in hybrid format, i.e., both in person and video calls, responded "Strongly agree" or "Agree," while only 1.5% of patients responded either "Strongly disagree" or "Disagree." Similarly, when patients were asked if they received the care they expected to receive upon consultation, i.e., if their expectations were met, 98% of the patients who attended in-person consultations responded "Strongly Agree" or "Agree." When patients attended consultations via video call or hybrid format, 91% responded, "Strongly agree" or "Agree." When asked if they found it tiring to have all consultations carried out in one day, 38% of patients who attended in-person consultations responded "Strongly agree" or "Agree." In contrast, 43% responded "Strongly disagree" or "Disagree."

## Discussion

The paper presents clinical indicators (e.g., HbA1c, blood pressure, BMI) and patient-reported outcomes (e.g., anxiety, depression, diabetes distress, quality of life), offering a balanced evaluation of the program's effectiveness. This paper also reports positive trends in glycemic control, blood pressure management, and obesity rates, indicating that the CAIPaDi program may positively impact these important health parameters. The low occurrence of acute complications, hypoglycemia, and no reported cases of lower limb amputations, deaths, or ischemic heart disease/congestive heart failure is a positive outcome, suggesting the safety of the program.

The CAIPaDi program provides a multidisciplinary approach to patients focused on maintaining metabolic control to diminish long-term complications. Given that patients require holistic treatment, using ICHOM's standardized set of outcomes and outcomes measurement tools ensures that the patient's needs are covered in every consultation.

Changes in glycemic control notably improved at three months and slowly decreased in annual evaluations. This could be attributed to pharmacological and non-pharmacological treatment modifications made at the monthly visits with their healthcare professionals. To avoid serious potential complications from severe hypoglycemia, treatment should be tailored individually so that hypoglycemia rates remain as low as possible.

Patient-reported outcomes measures reported emotional and psychosocial wellbeing and overall quality of life. This is attributable to consultations with psychology experts during visits and improved overall wellbeing related to disease and symptoms.

Psychosocial and emotional wellbeing, therefore, greatly benefits from an integral and comprehensive approach, such as the one implemented in CAIPaDi, as patients receive medical care and attention focused on all aspects of their health, which may be affected by diabetes and also because they feel better informed and in control of their diagnosis and treatment. Patients found the questionnaires easy to complete and felt their expectations of the medical attention received were properly met, which is an important factor in patient experience and satisfaction, benefitting overall wellbeing.

The key elements of success and lessons learned are that results observed in patient outcomes can be attributed to several factors. First, visits have been standardized and systematized. As such, all patients go through the same care pathway, and all professionals must cover all aspects of consultation. These services include endocrinology, focusing on diabetes; patient education; nutrition and physical activity; clinical psychology; dentistry, psychiatry; ophthalmology, focusing on retina and optometry for patients with diabetes; and podology, focusing on conditions secondary to diabetes. When a patient visits the practice, be it for an initial or follow-up consultation, they are seen by health professionals from each of these services and follow the same order to ensure an integrated and structured assessment of the patient is carried out. This minimizes the risk of underdiagnosis or misdiagnosis and the risk of developing complications and deterioration of the patient's condition. Through this model, health professionals attend to patients rather than isolated conditions.

Meanwhile, this standardized care model allows patients to be assessed through an individualized lens, which is essential to achieving results and enhancing patients' adherence to treatment. Additionally, measuring mental health-related outcomes, a key aspect of patients' overall wellbeing, is prioritized equally as other clinical outcomes. As most mental health-related outcomes are measured through patient-reported tools, patients can give their perspective in a way that can be objectively measured by health professionals, facilitating the need for referrals and respective decision-making. Data is continually collected and analyzed, which allows a consistent evaluation of results. This allows for continuous improvement of the healthcare services and the outcome measurement process and encourages the acknowledgment of the impact on patient's wellbeing through this model. Also, rapid diagnostics are key to the overall improvement in program adherence. Finally, the investment in electronic tools to optimize data collection is key, as it will allow for a more feasible implementation of the outcome measurement process, notably through data and electronic medical records software.

Ensuring patients' adherence to treatment is a fundamental aspect of achieving optimal results through this and any healthcare program. Patient education is one of CAIPaDi's standardized elements of consultation, and this is a crucial moment to enhance patients' engagement in the program and ensure they are adequately informed about their condition, their treatment, and all its implications. Adequate patient education will improve patients' autonomy and joint decision-making and boost their engagement, increasing their likelihood of attending follow-up consultations and adhering to their prescribed treatment. Further, it is indispensable that all health professionals adhere properly to the program. Sufficient institutional resources must be dedicated to the program's needs, including staff, time, material, and monetary resources. Similarly, all health professionals must be fully engaged and adhere to the program's structure for the integrated, comprehensive execution of the model to be successful.

A key objective will be reducing the difference between the number of patients at the first and last visits, increasing adherence to treatment among patients, and increasing engagement from the health professional's side to the program.

The study has strengths and limitations. Strengths include the longitudinal analysis covering five years and providing a comprehensive view of patient outcomes over an extended duration, which allows for a more robust understanding of the impact of the CAIPaDi program. Patient eligibility criteria encompass various factors, including demographic, social, behavioral, and health status, ensuring a holistic assessment of patient health and wellbeing.

The main limitation is that it needs to be more generalizable to a broader population, as it only includes patients enrolled in the CAIPaDi program. It may not represent the wider population with diabetes or other types of diabetes different from type 2. Some variables need to be registered from the beginning. This limitation has changed, and we have now registered them systematically. There is a high percentage of program abandonment, and we could not register outcomes from that group. Another limitation is that the education level of the patients should have been considered in the analytical model.

## Conclusion

The CAIPaDi model has established a framework that allows for a multidisciplinary approach to assess patients in an integrated and standardized manner, allowing for more beneficial and individualized patient care. Increasing patient engagement and adherence to treatment and encouraging them to continue to show up for follow-up will improve their outcomes even further in a way that is meaningful and impactful to them as patients living with diabetes.

## Data Availability

The data that support the findings of this study are not openly available due to reasons of sensitivity and are available from the corresponding author upon reasonable request.

## Abbreviations

ACE: Angiotensin-converting enzyme
ACR: Albumin-creatinine ratio
BMI: Body mass index
BP: Blood pressure
CAIPaDi: Centro de Atención Integral al Paciente con Diabetes (Center of Comprehensive Care for Patients with Diabetes)
CHF: Chronic heart failure
CKD-EPI: Chronic Kidney Disease Epidemiology Collaboration
CROs: Clinically reported outcomes
CV: Cardiovascular
DKA: Diabetic ketoacidosis
DPP IV: Dipeptidyl peptidase 4
DQoL: Diabetes Quality of Life
ER: Emergency room
GLP1: Glucagon-like peptide 1
HbA1c: Glycated hemoglobin
HHS: Hyperglycemic hyperosmolar state
ICHOM: International Consortium for Health Outcomes Measures
IHD: Ischaemic heart disease
LDL-c: Low-density lipoprotein-cholesterol
T2D: Type 2 diabetes
PAD: Peripheral artery disease
PAID: Problem Areas In Diabetes
PHQ-9: Patient Health Questionnaire
PRO: Patient-reported outcomes
PROM: Patient-reported outcomes measures
SD: Standard deviations
SGLT2: Sodium-glucose transporters
SMID: Sistema de Monitoreo Integral en Diabetes (Comprehensive Diabetes Monitoring System)
UTI: Urinary tract infection
WHO-5: World Health Organization questionnaire
